# Functional profiles of curatively treated adenoid cystic carcinoma unveil prognostic features and potentially targetable pathways

**DOI:** 10.1038/s41598-023-28901-9

**Published:** 2023-01-31

**Authors:** Chiara Romani, Luigi Lorini, Anna Bozzola, Eliana Bignotti, Michele Tomasoni, Laura Ardighieri, Mattia Bugatti, Simonetta Battocchio, Antonella Ravaggi, Davide Tomasini, Marco Ravanelli, Cristina Gurizzan, Davide Lombardi, Davide Mattavelli, Stefano Calza, Cesare Piazza, Paolo Bossi

**Affiliations:** 1 Angelo Nocivelli Institute of Molecular Medicine, University of Brescia and ASST Spedali Civili di Brescia, Brescia, Italy; 2 Department of Medical and Surgical Specialties, Radiological Sciences and Public Health, University of Brescia, Brescia, Italy; 3 Medical Oncology Unit, ASST Spedali Civili di Brescia, Brescia, Italy; 4 Department of Pathology, ASST Spedali Civili di Brescia, Brescia, Italy; 5 Unit of Otorhinolaryngology-Head and Neck Surgery, ASST Spedali Civili di Brescia, Brescia, Italy; 6 Department of Radiation Oncology, ASST Spedali Civili di Brescia, Brescia, Italy; 7 Unit of Biostatistics and Bioinformatics, Department of Molecular and Translational Medicine, University of Brescia, Brescia, Italy

**Keywords:** Head and neck cancer, Cancer genomics, Oncology

## Abstract

Adenoid cystic carcinoma (ACC) of salivary gland is a slowly growing tumor showing a propensity for delayed recurrence, with decreased survival rates. The identification of poor prognosis patients may help in defining molecular-based targeted strategies in this rare disease orphan of new treatments. Through a gene expression microarray-based approach followed by GSE functional analysis the expression profile of 46 primary untreated ACC samples and of ACC (h-TERT) tumor cells was analyzed. Patients who experienced early relapse showed enrichment in proliferation-related gene sets, including the G2-M checkpoint, E2F and myc targets, and in gene sets related to IFN signaling and aberrant proteostasis (FDR < 0.1), indicating increased mitotic and transcriptional activity in aggressive ACC. Similar functions were enriched in ACC samples classified by immunohistochemical staining as p63-negative, which exhibited increased protein burden and activation of pro-survival stress response pathways compared to p63-positive tumors. Compared to ACC tissues, ACC (h-TERT) cells share transcriptional features of aggressive p63-negative tumors. These data suggest association of specific pathway alterations with histopathological features of ACC, as recapitulated by p63 testing in patient prognostic stratification, anticipating new avenues for therapeutic intervention.

## Introduction

Adenoid Cystic Carcinoma (ACC) is a rare cancer, representing 1% of all Head and Neck Cancers (HNC) and 20% of all Salivary Gland Cancers (SGC)^1^, with a reported incidence of 4.5 cases/100,000 individuals^2^. It is generally considered as a long-term poor prognosis tumor with survival rates at 5,10, and 20 years around 68%, 52% and 28% respectively^3^. Despite advances in the gold standard treatment for the locoregional disease, represented by surgery and radiotherapy (RT), more than 75% of patients with locally advanced disease experience relapse, and from 8 to 60% of patients, according to different studies, develop distant metastasis^4,5^.

Among the reasons for this poor prognosis is the lack of effective clinical markers, informative of the clinical evolution of the disease, and/or predictive of treatment response, that could pave the way to more tailored therapeutic approaches. Moreover, despite advances in understanding the ACC underlying molecular alterations, tumor biology remains actually largely unknown, and the application of genomic predictors in clinical settings is still limited. The comprehensive genomic analysis of ACC samples has shown few recurrent mutations in known genes and a substantially low mutational burden, confirming the MYB/MYBL1-NFIB fusions as key events in the pathogenesis of ACC and a hallmark of this pathology, being present in more than 50% of cases^6,7^. Among the prognostic markers and potential therapeutic targets, the NOTCH mutation, reported in 15–20% of tumors, has been associated with advanced stages, solid histology, and shorter Overall Survival (OS), the latter only in univariate analysis^8^. Recently, a multidimensional analysis integrating mutational, transcriptional and proteomic data, highlighted the existence of two ACC molecular subtypes, ontogenetically distinct, with multiple and unique molecular alterations in each subtype, providing opportunities for combinational therapies^9^.

While histological and clinical characteristics could not totally describe the behaviour of the disease, a more in-depth molecular and biological analysis of this neoplasm might help both in depicting prognostic information and finding new potential therapeutic targets. The main aim of our study is to evaluate prognostic features and potential targetable pathways using gene expression analysis in a series of curatively treated ACC.

## Patients and methods

### Patient information

We included all consecutive treatment naïve patients diagnosed and treated with curative intent for ACC at the Unit of Otorhinolaryngology-Head and Neck Surgery, ASST Spedali Civili di Brescia, Italy, from 1996 to 2019. Eligible patients had a histological diagnosis of ACC, they were primarily treated with surgery, they had a histological sample available for tissue analysis and an adequate follow up. Patients could receive postoperative radiation, according to clinical stage and pathological characteristics. Written informed consent was obtained from all patients enrolled as per protocol approved by the Research Review Board-the Ethic Committee- of the ASST Spedali Civili, Brescia, Italy (study reference number: NP4266). The clinical and follow-up information were recorded from patients’ files. Clinical stage was re-assessed according to the 8th edition of the AJCC classification.

### Sample collection and histopathological analysis

Paraffin blocks from ACC tissue were generated at the time of surgery, from the primary tumor. All cases were reviewed by two expert head and neck pathologists (A.B. and L.A.) and the diagnosis were confirmed using H&E staining. Histologic grading was obtained by evaluating growth patterns, the percentage of solid areas and the cytological characteristics of the neoplastic cells. Carcinoma mostly tubular with some cribriform patterns, with the absence of solid areas or nuclear pleomorphism and low mitotic activity were classified as grade 1; cases with pure cribriform or mixed tubular/cribriform patterns, solid areas lower than 30% and slightly greater degree of nuclear pleomorphism and mitotic activity were classified as grade 2; cases with solid pattern greater than 30%, with a more significant nuclear pleomorphism, mitotic activity and necrosis were classified as grade 3^10^. Immunohistochemical analysis with p63 monoclonal antibody (DAK-63; Agilent) through Leica Bond III staining platform was performed on serial 5 µm sections of selected FFPE representative tumor blocks. P63 expression was considered positive when uniform or diffuse strong nuclear staining was observed in > 70% of tumor cells, as previously described^9^.

### RNA extraction and gene expression profiling

Total RNA was isolated with the RNeasy DSP FFPE Kit (Qiagen) according to the manufacturer’s instructions. RNA concentration and 260/280 absorbance ratio (A260/280) was measured with the Infinite M200 spectrophotometer (TECAN) and quality control of extracted RNA assessed with RT-qPCR for 18S rRNA. RNA from ACC (h-TERT) cells was kindly gifted by Prof. Sandra Sigala (University of Brescia).

To generate gene expression profiles, total RNA was converted to cRNA and then to cDNA, and hybridized, according to the manufacturer’s guidelines, on the Human Clariom S GeneChip (ThermoFisher Scientific), optimized for FFPE samples and able to accurately measure gene-level expression from > 20,000 well-annotated genes. The Scanner 3000 7G (ThermoFisher Scientific) was used in conjunction with GeneChip Operation Software (ThermoFisher Scientific) to generate one CEL file per hybridized cDNA.

### Microarray data processing and analysis

Arrays underwent quality control before being processed with RMA algorithm^11^. Hierarchical clustering was performed using Euclidean distance metric and Ward’s agglomerative algorithm^12^. Genes were filtered based on association with DFS evaluated using gene-level multivariable Cox models. Top genes were selected based on unadjusted p-value less than 5%. Differential expression (DE) between groups was modelled using linear models with t-statistic computation based on Empirical Bayesian algorithm (limma)^13^. Filtered features were further processed to evaluate potential genesets enrichment using Gene Set Enrichment Analysis (GSEA)^14,15^ and both Hallmark^16^ and GO geneset lists.

An enrichment score for selected genesets (hereafter global score) for each patient were computed using single sample GSEA (ssGSEA)^17^ which computes an enrichment score for every subject-geneset based on a non-parametric approach: such score can be interpreted as the level of global expression of a specific geneset for every subject.

### Statistical analysis

Data were described using mean (standard deviation) and median (Interquartile range, IQR) for quantitative variables and percentages for categorical.

Association between categorical variables was evaluated using a Chi-squared test with p-value computed using Monte Carlo simulation (B = 2000).

Disease-free survival (DFS) was modelled as a function of clinical variables using Cox models. Follow-up median time was estimated using the Kaplan–Meier method. Optimal cutpoints for continuous variables, as predictors of time-to-event outcomes, were computed using maximally selected log-rank statistics^18^. All tests were two sided and assumed a significance level of 5%. All analyses were performed using R (version 4.1.1).

### Institutional review board statement

The study was conducted according to the guidelines of the Declaration of Helsinki and approved by the Institutional Review Board of ASST Spedali Civili, University of Brescia, Italy (study reference number: NP4266).

## Results

### Clinical and pathological description of ACC cohort

We identified 83 patients with ACC matching inclusion criteria, whose clinical and pathological characteristics are summarized in Table 1. Mean age at diagnosis was 53.4 years (median 53.5, range [21–85]) and gender was predominantly female (67%). In 74% of cases ACC developed from minor salivary glands. At diagnosis, 77% of tumors were classified as T3–4, 88% had no nodal involvement, and 70% of the patients with nodal involvement presented extranodal extension. Adjuvant radiotherapy was performed in 81% of the patients. During follow-up, 29 patients died (35%) and 42 (51%) had a disease relapse; median follow-up time was 124 months (IQR 61–167), while median time to relapse was 77 months. Overall, 37 patients were excluded from subsequent analysis for either poor quality of FFPE tumor block (N = 5) or poor quality of extracted RNA (N = 32). Characteristics of the 37 patients excluded were compared to those of eligible patients, to investigate possible selection bias. No significant differences were found (Supplementary Table S1).

**Table 1:** Patient cohort description.

	Overall ACC cohort n (%)	Gene expression study ACC cohort n (%)
Number of patients	83 (100%)	46 (55%)
Gender		
Female	56 (67)	35 (76)
Male	27 (33)	11 (24)
Age at presentation (years)		
Median	53.5	51.0
Range	(21–85)	(21–85)
pT		
pT1	10 (12)	5 (11)
pT2	6 (7)	3 (7)
pT3	14 (17)	10 (22)
pT4	50 (60)	27 (59)
Miss	3	1
pN		
−	73 (88)	41(89)
+	10 (12)	5 (11)
ENE		
−	3 (30)	2 (40)
+	7 (70)	3 (60)
Grade		
I–II	–	35 (76)
III	–	11 (24)
Site		
Major	22 (26)	14 (30)
Minor	61 (74)	32 (70)
Adjuvant RT		
No	15 (18)	12 (26)
Yes	67 (81)	33 (72)
Miss	1	1
Recurrence at last follow up		
No	41 (49)	19 (41)
Yes	42 (51)	27 (59)
Dead at last follow up		
No	54 (65)	29 (63)
Yes	29 (35)	17 (37)

*ENE* extra nodal extension, *RT* radiotherapy, *N* lymph node.

Pathological grading was performed on H&E sections of the 46 ACC patients eligible for gene expression profiling. As shown in Table 1, 35 out of 46 patients (76%) were classified as grade 1/2, while 11 cases were classified as grade 3 (24%).

Survival analysis of clinical and pathological risk factors for local–regional recurrence or distant metastasis indicate that T class, histological grade, and adjuvant RT have prognostic impact on DFS, with advanced stage and high grade independently associated with reduced DFS (Table 2).

**Table 2:** Disease-free survival analysis for ACC patients (n = 83). Clinical and pathological risk factors for local–regional recurrence or distant metastasis were analyzed using Multivariable Cox’s proportional-hazards models.

Characteristics	DFS			
	Univariate		Multivariate	
	HR (95% CI)	p value	HR (95% CI)	p value
Gender				
Male vs female	1.72 (0.92–3.24)	0.09	1.49 (0.77–2.88)	0.293
T stage				
T3–4 vs T1–2	2.64 (0.94–7.41)	0.065	4.69 (1.41–15.6)	0.012
Histological grade				
G3 vs G1–2	3.92 (1.72–8.94)	*0.001	4.92 (1.86–13)	*0.001
Site				
Major vs Minor	1.12 (0.56–2.22)	0.754	0.90 (0.43–1.87)	0.778
Adjuvant RT				
Yes vs no	0.75 (0.35–1.63)	0.472	0.30 (0.12–0.78)	0.013

*HR* hazard ratio, *CI* confidence interval.

*n = 46.

### Different biological processes are associated with prognosis in ACC

**Figure 1:**
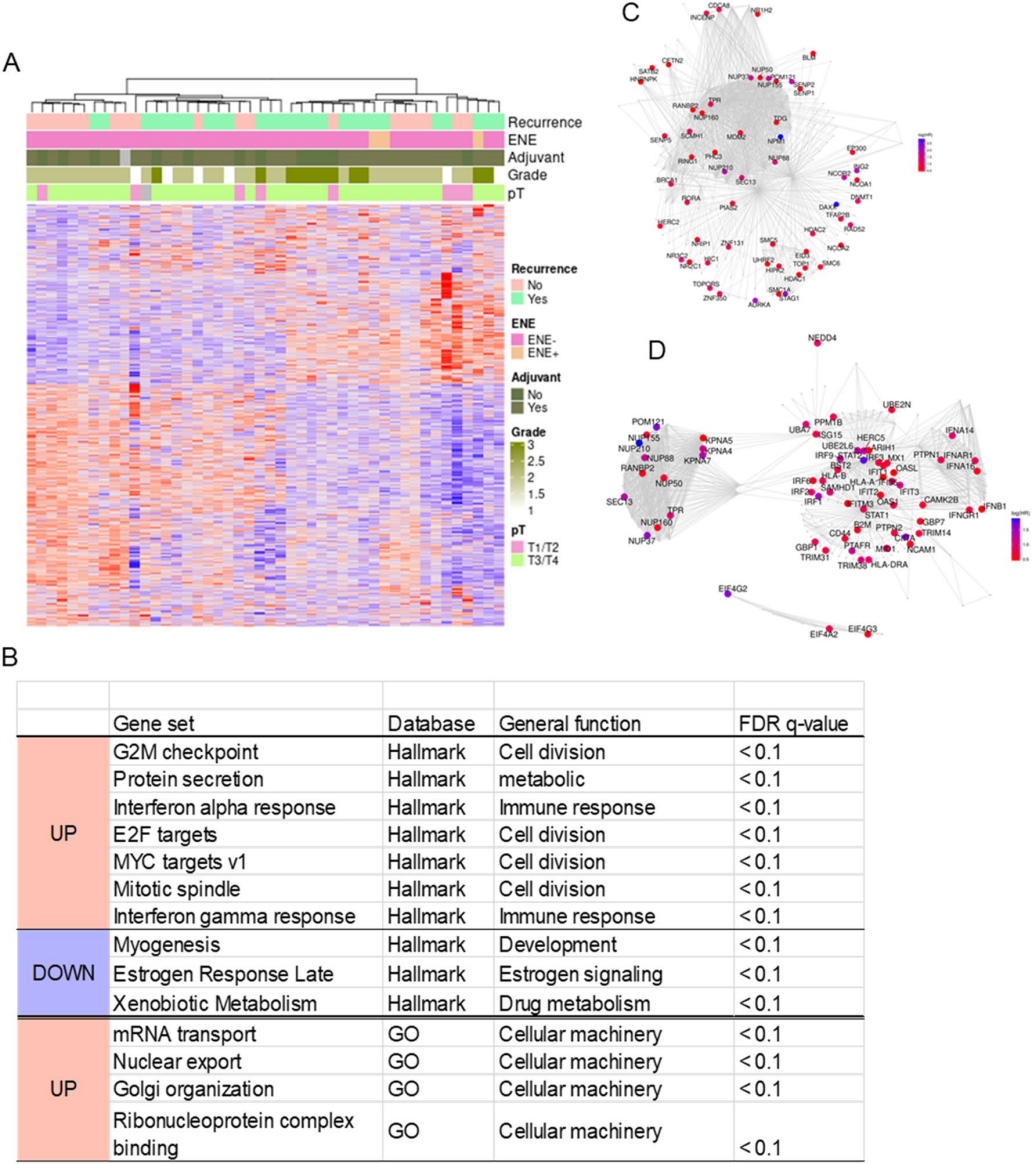
Transcriptional profiling to predict prognosis of ACC patients. (**A**) Heatmap for hierarchical clustering of relevant genes selected based on DFS hazard ratio (*p* value < 0.05). Clinicopathologic ACC features shown in the upper panels. (**B**) Pathways enriched in patients with short DFS by GSEA with Hallmark and GO genesets. (**C**,**D**) REACTOME pathways for SUMOylation and Interferon Signaling. Color band displays the log (HR) value for the leading-edge genes in each pathways.

The global gene expression profile of ACC samples was generated by microarray platform, to obtain a comprehensive picture of transcriptomic landscape characterizing relapsing patients. Analysis was performed at gene-level using multivariate Cox models. Of the ~ 20,000 genes present on the chip, we found 650 genes significantly associated with DFS (p-value < 5%). Hierarchical clustering of the selected genes identified two distinct clusters with no evident enrichment for advanced stage tumors, grade or specific subsites nor difference according to adjuvant treatment strategy among the subgroups (Fig. 1A).

Using Hallmark collection, GSEA shows that the top genesets positively associated with shorter DFS are G2-M checkpoint genes, mitotic spindle, E2F and myc targets (all FDR < 0.1) These are largely recapitulated by GO functional enrichment analysis, which indicates cell cycle regulation, protein synthesis and ribosome genesis pathways significantly up-regulated in patients with early relapse (all FDR < 0.1) and collectively suggest that ACC progression is marked by both an increased mitotic activity and rates of protein synthesis. Moreover, the up-regulation of Interferon alpha and gamma response genesets in poor-prognosis patients indicates increased expression of target genes in response to IFN stimulation (Fig. 1B, Supplementary Table S2). The top down-regulated genesets include xenobiotic metabolism, estrogen response and myogenesis (all FDR < 0.1), the latter highlighting the loss of myoepithelial component in aggressive tumors (Fig. 1B). REACTOME analysis shows a similarity of enriched pathways in high-risk ACC, confirming a dysregulation of cell cycle and IFN signaling, and an increase in SUMOylation, a key post-translational modification associated with chromatin remodeling which play stress-mitigation function under strong oncogenic stimuli (FDR < 0.05, Fig. 1C,D, Supplementary Table S2).

### Defining ACC molecular subgroups with different prognosis

**Figure 2:**
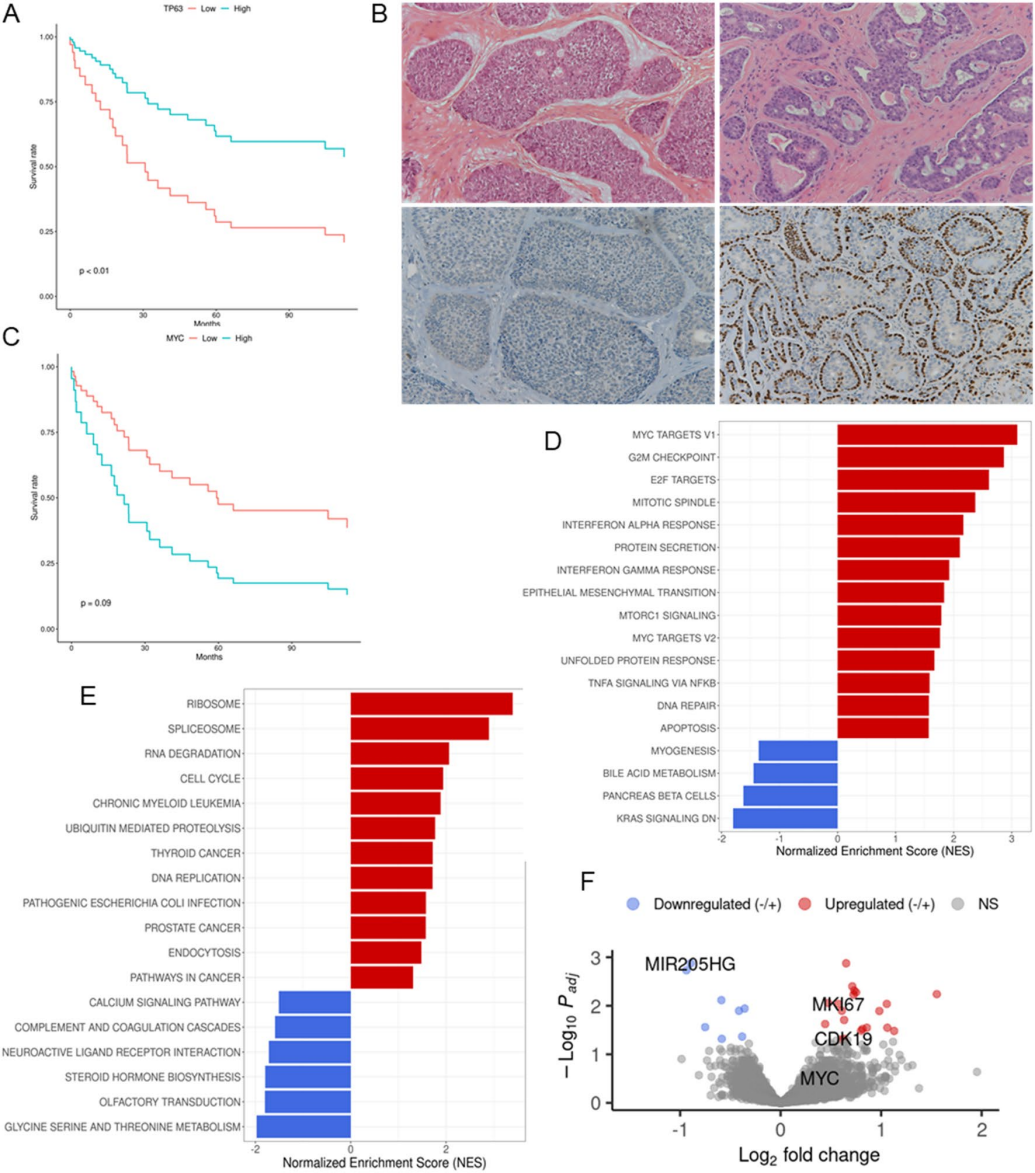
Distinct transcriptional features are associated with p63-negative ACC. (**A**–**C**) Kaplan–Meier curves showing the DFS as a function of TP63 or MYC mRNA expression. p-value computed from Likelihood Ratio Test (LRT). (**B**) Representative cases of ACC showing either diffuse strong nuclear p63 immunostaining in more than 70% of tumor cells (low right panel) or completely negative p63 expression (low left panel). Corresponding H&E sections are shown in the upper panel (10 × magnification). (**D**,**E**) Functional enrichment analysis depicting biological processes up-regulated in p63^neg^ ACC group compared to p63^pos^ tumors, considering both Hallmark and KEGG genesets. Each distribution represents the t-statistic associated to the core-enrichment genes of each pathway. (**F**) Volcano plot of genes with increased expression in p63^neg^ (red dots) and p63^pos^ (blue dots) ACCs.

A molecular subtyping of ACC centered on TP63 expression has been proposed as opposed to constitutive activation of MYC induced by NOTCH mutation/pathway alteration, both indicated as drivers of unique regulatory programs in ACC, with direct prognostic implications^9^. Following this concept, we evaluated patients' survival as a function of TP63 mRNA expression level, whose optimal cut-off value was computed using maximally selected statistics, and we observed a significantly reduced DFS in patients with low TP63 expression (HR: 0.34, CI_95%_ 0.15–0.77, *p* = 0.01, Fig. 2A). This prognostic relevance is independent of tumor stage and adjuvant treatment but strongly correlated to histological grade. Indeed, immunohistochemistry confirmed negative p63 expression in almost all grade 3 cases (9/11, 82%), with only two high grade cases revealing strong and diffuse expression of p63 in solid areas, whereas all grade 1–2 ACC showed positive p63 expression with a strong nuclear staining in > 30% of tumor cells (100% positive in grade I and II vs 18% in grade III, Chi-squared test p-value < 0.01) (Supplementary Table S3, Fig. 2B). At functional level, these data are consistent with the down-regulation of myogenesis pathway observed in early relapsing patients, and indicate p63 loss as a predictor of unfavourable outcome.

We then performed survival analysis according to MYC mRNA expression. Although with marginal statistical significance, evident survival differences were observed between patients in high and low MYC score (HR: 2.60, CI_95%_ 0.94–7.15, *p* = 0.065, Fig. 2C). Of interest, exploring MYC expression in our cohort of patients classified as p63-positive (p63^pos^) or p63-negative (p63^neg^) according to IHC staining, we found an increase of myc mean expression in p63^neg^ samples (FC = 1.311, p = 0.008, Supplementary Table S3). GSEA based on Hallmark collection, show that cell-cycle and proliferation related genesets, such as MYC targets, G2M checkpoint and E2F targets were significantly enriched in the p63^neg^ tumors, along with protein secretion and unfolded protein response (UPR), a network of signaling pathways that respond to stress in the endoplasmic reticulum (ER), and referred to as important mediators in maintaining cell survival in stressed conditions (all FDR < 0.05). MTORC1 signaling and DNA damage, two closely related pathways that communicate to face metabolic and xenotoxic stresses, were also enriched in the p63^neg^ group, together with the immune-related pathways interferon gamma/alpha responses. The RAS signaling, composed of genes that are downregulated when the KRAS signaling pathway is activated, is among the most significantly enriched pathways in p63^pos^ ACCs (Fig. 2D). KEGG pathway analysis^19–21^ shows consistent enrichment in cell cycle, ribosome and spliceosome functions and increase ubiquitin-mediated proteolysis in p63^neg^, overall suggestive of a compromised metabolic environment (Fig. 2E).

Among genes with the highest differential expression between p63^pos^ and p63^neg^ ACC we shortlisted the proliferation marker MKI67 and the cyclin dependent kinase CDK19, both highly upregulated in p63^neg^ tumor (FC > 1.2, FDR < 0.05, p < 0.001). MIR205HG, a long non-coding RNA enriched in the basal layer of epithelia and whose expression is regulated by p63, was among the highest up-regulated genes among the p63^pos^ (FC > 1.2, FDR < 0.05, p < 0.001) (Fig. 2F, Supplementary Table S4).

### The transcriptional profile of aggressive ACC is recapitulated in cell model of the disease

We exploited microarray data to link ACC transcriptomics with pharmacological information previously obtained in ACC (h-TERT) tumor cells^22^, a well-established experimental model of ACC with epi-myoepithelial derivation, lacking p63 expression^23^. By comparing the expression profile of ACC patients to cells, we found that the transcriptional profile of ACC (h-TERT) resembles that of ACC recurrent patients and indirectly links to myc expressing (Supplementary Fig. S1), suggesting that this molecular profile is maintained also when cancer cells are cultured in vitro, and collectively indicating ACC (h-TERT) as a cell model of aggressive ACC.

## Discussion

The clinical behavior of ACC is not straightforward, as many patients with locally advanced disease show a relapse, but with a different timing of recurrence, disease aggressiveness, type of relapse and response to treatments. This unpredictable clinical course is mirrored by the lack of strong pathological and molecular data, which do not fully capture the extent of heterogeneity present in this tumor. Herein, we exploited microarray-generated gene expression data to identify functional profiles associated with poor disease-free survival in curatively treated ACC, in the attempt to clarify the molecular mechanism driving its propensity to recur and to provide additional information to current prognostic assessment. By integrating transcriptomic alteration with patient clinical and pathological information, we demonstrated the prognostic relevance of multiple signaling pathways altered in ACC, extending the vision on the molecular processes mediating ACC biological aggressiveness beyond mutational mechanism^24–26^. These include the downregulation of the myogenesis pathway and the enrichment of proliferation and cell-cycle related processes such as myc targets activation and increased expression of Ki-67 mRNA, as well as increased protein burden and signs of energetic stress. Of note, functional analysis of samples clustered based on p63 immunostaining revealed highly consistent pathway alterations in p63-negative tumors and in early relapsing patients, suggesting a link between molecular events underlying the pathway p63 negative (mostly solid tumor and with high Ki67) and ACC adverse outcome. Together these results are in agreement with Ferrarotto and colleagues^9^ and functionally reflect the good prognosis of p63-positive ACC type II and the poor-prognosis of myc-driven ACC type I. An increased level of UPR signaling observed in p63*-*negative ACCs marks the occurrence of ER stress in this tumor subgroup and provides further connection with myc oncogenic activation^27^. This pro-survival stress response pathway, which helps cells to adapt to the higher demand of protein synthesis to sustain oncogenic-driven unrestricted cell proliferation, has also been implicated in chemotherapy resistance in several tumors, including breast and colorectal cancers^28,29^ through a mechanism that remains poorly understood, but that at the same time could pave the way towards new therapeutic application for chemo and radiotherapy refractory patients. For instance, small-molecule UPR inhibitors that directly target UPR sensor, such as IRE1alpha, have demonstrated antitumor activity and enhanced response to chemotherapy in preclinical model of triple negative breast cancer and pancreatic neuroendocrine tumors^30,31^. Along with the global increase of transcriptional activity, ER stress was also reported to enhance chromatin accessibility in activated genes^32^, suggesting the occurrence of epigenetic deregulation during ACC progression and potentially anticipating new avenues for therapeutic intervention.

Beside key alterations involving cell cycle related functions, pathway analysis indicates an activation of endogenous IFN signaling in aggressive ACC tumors. At odds with the well-recognized pro-apoptotic, pro-inflammatory, antitumor role of IFNs several lines of evidence raised in support of the pro-tumoral properties of this cytokine^33,34^ and the activation of IFN signaling in tumor cells has been linked to an unfavorable response to DNA damage intervention, including radiation^35^. In this regard, specific signatures of IFN-stimulated genes were traced in breast cancer patients and correlated with radiotherapy resistance and poor prognosis^36^.

Of note, the transcriptional profile of aggressive ACC is recapitulated in the cell model of the disease. Indeed, when compared with tumors, ACC (h-TERT) cells reveal the molecular features of aggressive ACC and look transcriptionally representative of patients with poor prognosis. As recently published by Savarese and colleagues testing ACC (h-TERT) cells with targeted agents show poor efficacy for either multikinase inhibitor lenvatinib and PI3-K inhibitor everolimus^22^. This finding is in line with the downregulation of RAS signaling pathways observed in poor prognosis patients and consistent with the upregulation of receptor tyrosine kinases genes in p63-positive ACC type II reported by Ferrarotto^9^, highlighting the importance of molecular screening for combination treatments, that currently does not represent a selection criterion for tailored treatments. Besides, our data strengthen the usefulness of p63 assessment for predicting biological behavior as p63 might represent a more reliable and reproducible marker than histopathological grading, which is collinear with other clinical pathological factors and suffers from interobserver variation^37^. Further studies in a large series of ACCs are warranted to confirm the potential of p63 as a prognostic marker as well as molecular marker for patient stratification in clinical trials.

From the technical standpoint, we proved the feasibility of a microarray-based transcriptional approach on FFPE samples, yet with a moderate successful rate as less than 40% of the retrospective cases turned out to be analyzable for poor quality of purified RNA. However, in our cohort, this appears not related to the archival time of clinical samples but rather to specific protocols required for routine diagnostic practice which may have a detrimental impact on nucleic acid quality.

A limitation of this study is represented by the lack of mutational data, specifically regarding the NOTCH status. Furthermore, we critically recognize that the lack of validation on an independent external cohort limits the generalizability of our findings. Still, results from pathway analysis are highly concordant to that recently published on fresh-frozen primary ACC, where a myc overexpressing subtype enriched in NOTCH activating mutations and associated with worse prognosis emerged from clustering by RNAseq and protein expression analysis^9^. Our work adds to this context by suggesting an association between specific transcriptional alterations and histopathological features of ACC, providing biological information relevant to the survival of ACC patients and new therapeutic perspectives.

## Supplementary Information

Supplementary Figure S1.

Supplementary Tables.

## Data Availability

Gene expression data are deposited at GEO DataSets with ID GSE214969 (https://www.ncbi.nlm.nih.gov/geo/query/acc.cgi?acc=GSE214969). The access is restricted to *reviewers* using the following token: cxgroeyandmbhwd. No other information is needed for accessing the data. The data will be made readily made publicly accessible once the paper is accepted for publication.
