# Supplementary material for: Functional profiles of curatively treated adenoid cystic carcinoma unveil prognostic features and potentially targetable pathways
Source: Sci Rep. 2023 Jan 31;13:1809. doi: 10.1038/s41598-023-28901-9 (PMC9889376; doi:10.1038/s41598-023-28901-9)

**Figure S1.** Clustering of gene expression data from ACC patients and ACC(h-TERT) cells.  The top color bar shows the patient's recurrence status and cell line sample. According to its gene expression profile the cell line clusters within the red group.


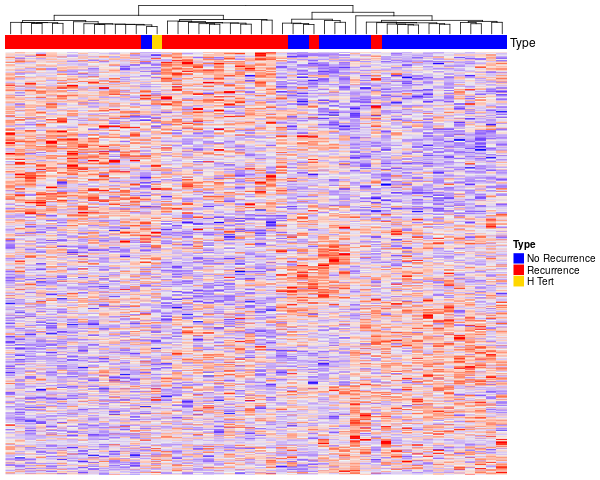

Supplement: Supplementary file 1 — Supplementary Figure S1. [file 41598_2023_28901_MOESM1_ESM.docx]
