# Supplementary material for: Functional profiles of curatively treated adenoid cystic carcinoma unveil prognostic features and potentially targetable pathways
Source: Sci Rep. 2023 Jan 31;13:1809. doi: 10.1038/s41598-023-28901-9 (PMC9889376; doi:10.1038/s41598-023-28901-9)
Supplement: Supplementary file 2 — Supplementary Tables. [file 41598_2023_28901_MOESM2_ESM.docx]

**Table S1.**

|  | **ACC overall cohort**  **n (%)** | **ACC gene expression study included cohort**  **n (%)** | **ACC gene**  **expression study excluded cohort**  **n (%)** | **p** |
| --- | --- | --- | --- | --- |
| **Number of patients** | 83 (100) | 46 (55) | 37 (45) |  |
| **Gender**  Female  Male | 56 (67)  27 (33) | 35 (76)  11 (24) | 21 (57)  16 (43) | 0.070 |
| **Age at presentation (years)**  Median  Range | 53.5  (21 - 85) | 51.0  (21-85) | 55.4  (34-76) | 0.227 |
| **pT**  pT1  pT2  pT3  pT4  miss | 10 (12)  6 (7)  14 (17)  50 (60)  3 | 5 (11)  3 (7)  10 (22)  27 (59)  1 | 5 (13)  3 (8)  4 (11)  23(62)  2 | 0.636 |
| **pN**  -  + | 73 (88)  10 (12) | 41 (89)  5 (11) | 32 (86)  5 (14) | 0.380 |
| **ENE**  -  + | 3 (30)  7 (70) | 2 (40.0)  3 (60.0) | 1 (20)  4 (80) |  |
| **Adjuvant RT**  No  Yes  miss | 15 (18)  67 (81)  1 | 12 (26)  33 (72)  1 | 3 (8)  34 (92)  - | 0.081 |
| **Recurrence at last follow up**  No  Yes | 41 (49)  42 (51) | 19 (41)  27 (59)  - | 22 (59)  15 (41) | 0.160 |
| **Dead at last follow up**  No  Yes | 54 (65)  29 (35) | 29 (63)  17 (37) | 25 (67)  12 (33) | 0.925 |

ENE (Extra Nodal Extension); RT (radiotherapy); N (lymphnode)

**Table S2.** Functional enrichment analysis depicting biological processes up-regulated in high-risk ACC patients considering GO and Reactome genestes. Each distribution represents the log(HR) associated to the core-enrichment genes of each pathway.

Considering GO Terms we have overall 75 gene sets with FDR (q-value) < 10%:

| **Description** | **FDR** |
| --- | --- |
| SMALL MOLECULE CATABOLIC PROCESS | 0.020 |
| RNA EXPORT FROM NUCLEUS | 0.021 |
| ACTIVE ION TRANSMEMBRANE TRANSPORTER ACTIVITY | 0.021 |
| REGULATION OF CELL CYCLE G2 M PHASE TRANSITION | 0.021 |
| LIPID CATABOLIC PROCESS | 0.021 |
| HP EMG MYOPATHIC ABNORMALITIES | 0.030 |
| ESTABLISHMENT OF RNA LOCALIZATION | 0.030 |
| NUCLEOBASE CONTAINING COMPOUND TRANSPORT | 0.030 |
| HP POINTED CHIN | 0.030 |
| PROTEIN DEPOLYMERIZATION | 0.030 |
| RIBONUCLEOPROTEIN COMPLEX BINDING | 0.030 |
| HORMONE METABOLIC PROCESS | 0.030 |
| SPINDLE | 0.030 |
| REGULATION OF CELLULAR RESPONSE TO HEAT | 0.033 |
| NUCLEAR PORE | 0.033 |
| CORNIFICATION | 0.033 |
| PEPTIDE RECEPTOR ACTIVITY | 0.033 |
| CHROMOSOME CENTROMERIC REGION | 0.033 |
| ACTIVE TRANSMEMBRANE TRANSPORTER ACTIVITY | 0.033 |
| RETROGRADE VESICLE MEDIATED TRANSPORT GOLGI TO ENDOPLASMIC RETICULUM | 0.034 |
| TERPENOID METABOLIC PROCESS | 0.034 |
| HP ABNORMALITY OF FEMUR MORPHOLOGY | 0.034 |
| MRNA TRANSPORT | 0.041 |
| ACTIN FILAMENT DEPOLYMERIZATION | 0.043 |
| DIENCEPHALON DEVELOPMENT | 0.043 |
| RNA LOCALIZATION | 0.044 |
| REGULATION OF LIPID CATABOLIC PROCESS | 0.046 |
| CARDIAC NEURAL CREST CELL DIFFERENTIATION INVOLVED IN HEART DEVELOPMENT | 0.048 |
| CELLULAR RESPONSE TO HEAT | 0.048 |
| CELL CYCLE G2 M PHASE TRANSITION | 0.048 |
| BMP BINDING | 0.050 |
| NCRNA EXPORT FROM NUCLEUS | 0.050 |
| HP HEPATOSPLENOMEGALY | 0.051 |
| CILIARY BASAL BODY PLASMA MEMBRANE DOCKING | 0.051 |
| HP THIN UPPER LIP VERMILION | 0.051 |
| HP VERTEBRAL COMPRESSION FRACTURES | 0.051 |
| MONOCARBOXYLIC ACID TRANSMEMBRANE TRANSPORTER ACTIVITY | 0.051 |
| NUCLEAR EXPORT | 0.058 |
| ANTIGEN PROCESSING AND PRESENTATION | 0.058 |
| PROTEIN MODIFICATION BY SMALL PROTEIN REMOVAL | 0.059 |
| GOLGI ORGANIZATION | 0.059 |
| HP LIMB MUSCLE WEAKNESS | 0.059 |
| REGULATION OF HORMONE LEVELS | 0.059 |
| CELLULAR LIPID CATABOLIC PROCESS | 0.059 |
| PORPHYRIN CONTAINING COMPOUND METABOLIC PROCESS | 0.065 |
| HP OPEN MOUTH | 0.066 |
| SYMPORTER ACTIVITY | 0.066 |
| HP DISSEMINATED INTRAVASCULAR COAGULATION | 0.066 |
| HP COLON CANCER | 0.066 |
| HP CONJUGATED HYPERBILIRUBINEMIA | 0.067 |
| MULTICELLULAR ORGANISMAL MOVEMENT | 0.068 |
| HP ABNORMALITY OF THE NASAL TIP | 0.073 |
| PROTEIN POLYUBIQUITINATION | 0.074 |
| HP DECREASED FERTILITY IN FEMALES | 0.075 |
| HEME METABOLIC PROCESS | 0.078 |
| HP PERIPHERAL VISUAL FIELD LOSS | 0.082 |
| HP RENAL STEATOSIS | 0.083 |
| HP UPPER LIMB MUSCLE WEAKNESS | 0.083 |
| REGULATION OF PROTEIN DEPOLYMERIZATION | 0.083 |
| NEUTRAL LIPID METABOLIC PROCESS | 0.083 |
| CELLULAR HORMONE METABOLIC PROCESS | 0.083 |
| MEMBRANE DOCKING | 0.083 |
| CELLULAR MODIFIED AMINO ACID METABOLIC PROCESS | 0.083 |
| ORGANIC ACID TRANSPORT | 0.083 |
| HP CONE SHAPED EPIPHYSIS | 0.086 |
| HP THIN VERMILION BORDER | 0.086 |
| RIBOSOME BINDING | 0.088 |
| MITOTIC SPINDLE | 0.088 |
| HP ABNORMALITY OF MOUTH SHAPE | 0.088 |
| SECONDARY ACTIVE TRANSMEMBRANE TRANSPORTER ACTIVITY | 0.090 |
| HP BULBOUS NOSE | 0.095 |
| MRNA EXPORT FROM NUCLEUS | 0.095 |
| HP ABNORMAL MUSCLE FIBER MORPHOLOGY | 0.095 |
| MUSCLE CELL DEVELOPMENT | 0.098 |
| HP VENTRICULAR PREEXCITATION | 0.098 |

Considering REACTOME we have overall 40 gene sets with FDR (q-value) < 10%:

| **Description** | **FDR** |
| --- | --- |
| M PHASE | 0.000 |
| ANTIVIRAL MECHANISM BY IFN STIMULATED GENES | 0.000 |
| REGULATION OF MECP2 EXPRESSION AND ACTIVITY | 0.001 |
| INTERFERON SIGNALING | 0.001 |
| MITOTIC PROPHASE | 0.007 |
| SUMOYLATION | 0.007 |
| CLASS I MHC MEDIATED ANTIGEN PROCESSING PRESENTATION | 0.007 |
| FORMATION OF THE CORNIFIED ENVELOPE | 0.008 |
| MITOTIC PROMETAPHASE | 0.016 |
| METALLOPROTEASE DUBS | 0.046 |
| INTERACTIONS OF REV WITH HOST CELLULAR PROTEINS | 0.046 |
| NS1 MEDIATED EFFECTS ON HOST PATHWAYS | 0.046 |
| EXPORT OF VIRAL RIBONUCLEOPROTEINS FROM NUCLEUS | 0.046 |
| AURKA ACTIVATION BY TPX2 | 0.046 |
| REGULATION OF PLK1 ACTIVITY AT G2 M TRANSITION | 0.046 |
| ANCHORING OF THE BASAL BODY TO THE PLASMA MEMBRANE | 0.046 |
| MAPK6 MAPK4 SIGNALING | 0.046 |
| MITOTIC SPINDLE CHECKPOINT | 0.046 |
| RHO GTPASE EFFECTORS | 0.046 |
| ANTIGEN PROCESSING UBIQUITINATION PROTEASOME DEGRADATION | 0.046 |
| PYRIMIDINE SALVAGE | 0.051 |
| SUMOYLATION OF DNA DAMAGE RESPONSE AND REPAIR PROTEINS | 0.051 |
| MITOTIC G2 G2 M PHASES | 0.051 |
| MITOTIC METAPHASE AND ANAPHASE | 0.051 |
| NUCLEAR ENVELOPE BREAKDOWN | 0.051 |
| GENE SILENCING BY RNA | 0.052 |
| SEPARATION OF SISTER CHROMATIDS | 0.058 |
| ESR MEDIATED SIGNALING | 0.072 |
| CELL CYCLE CHECKPOINTS | 0.072 |
| METABOLISM OF PORPHYRINS | 0.075 |
| SYNTHESIS OF ACTIVE UBIQUITIN ROLES OF E1 AND E2 ENZYMES | 0.075 |
| SUMOYLATION OF DNA REPLICATION PROTEINS | 0.075 |
| RECRUITMENT OF MITOTIC CENTROSOME PROTEINS AND COMPLEXES | 0.075 |
| RAC3 GTPASE CYCLE | 0.075 |
| ESTROGEN DEPENDENT GENE EXPRESSION | 0.075 |
| CELLULAR RESPONSE TO HEAT STRESS | 0.075 |
| GPCR LIGAND BINDING | 0.075 |
| TRANSPORT OF MATURE MRNAS DERIVED FROM INTRONLESS TRANSCRIPTS | 0.076 |
| NUCLEAR IMPORT OF REV PROTEIN | 0.082 |
| HEPARAN SULFATE HEPARIN HS GAG METABOLISM | 0.094 |

**Table S3.** Immunohistochemical analysis with p63 monoclonal antibody performed on selected FFPE representative tumor blocks of ACC patients (n=46); % of tumor cells showing positive p63 nuclear staining are indicated. Information on histological grade and tumor site are reported.

| Tumor | Grade | p63 (%) | Tumor site |
| --- | --- | --- | --- |
|  |  |  |  |
| ADCC 63 | 1 | 100 | MAJ |
| ADCC 69 | 1 | 100 | MIN |
| ADCC 15 | 1 | 80 | MAJ |
| ADCC 88 | 1 | 100 | MAJ |
| ADCC 47 | 1 | 100 | MAJ |
| ADCC 53 | 1 | 100 | MIN |
| ADCC 66 | 2 | 100 | MIN |
| ADCC45 | 2 | 100 | MIN |
| ADCC 13 | 2 | 100 | MIN |
| ADCC70 | 2 | 100 | MIN |
| ADCC 71 | 2 | 100 | MIN |
| ADCC 72 | 2 | 100 | MIN |
| ADCC 73 | 2 | 100 | MAJ |
| ADCC75 | 2 | 100 | MAJ |
| ADCC 76 | 2 | 100 | MAJ |
| ADCC 107 | 2 | 90 | MIN |
| ADCC 48 | 2 | 100 | MIN |
| ADCC 5 | 2 | 100 | MIN |
| ADCC 77 | 2 | 100 | MAJ |
| ADCC 80 | 2 | 100 | MAJ |
| ADCC62 | 2 | 100 | MIN |
| ADCC68 | 2 | 100 | MIN |
| ADCC 81 | 2 | 100 | MIN |
| ADCC 4 | 2 | 100 | MAJ |
| ADCC 58 | 2 | 100 | MAJ |
| ADCC 33 | 2 | 100 | MIN |
| ADCC 20 | 2 | 100 | MIN |
| ADCC 26 | 2 | 100 | MIN |
| ADCC 6 | 2 | 100 | MIN |
| ADCC 105 | 2 | 100 | MIN |
| ADCC 50 | 2 | 100 | MIN |
| ADCC 25 | 2 | 75 | MAJ |
| ADCC 41 | 2 | 100 | MAJ |
| ADCC 102 | 2 | 100 | MIN |
| ADCC 28 | 2 | 100 | MIN |
| ADCC 44 | 3 | 10 | MAJ |
| ADCC 60 | 3 | 0 | MIN |
| ADCC 103 | 3 | 0 | MIN |
| ADCC 24 | 3 | 0 | MIN |
| ADCC 78 | 3 | 100 | MIN |
| ADCC 85 | 3 | 0 | MIN |
| ADCC 86 | 3 | 0 | MIN |
| ADCC 56 | 3 | 100 | MIN |
| ADCC 83 | 3 | 0 | MIN |
| ADCC7 | 3 | 0 | MIN |
| ADCC64 | 3 | 0 | MIN |

|  |  |  |  |
| --- | --- | --- | --- |
|  |  |  |  |
|  |  |  |  |
|  |  |  |  |
|  |  |  |  |

**Table S4.** Genes differentially expressed between p63^neg^ and p63^pos^ ACCs (FDR<0.05, p<0.001).

| Gene | FC | FDR | pvalue |
| --- | --- | --- | --- |
| COL17A1 | 0.378 | <0.01 | <0.001 |
| ADAMTS16 | 3.712 | <0.01 | <0.001 |
| MIR205HG | 0.538 | <0.01 | <0.001 |
| LRP6 | 1.571 | <0.01 | <0.001 |
| TNS4 | 0.522 | <0.01 | <0.001 |
| TUBA1B | 1.638 | <0.01 | <0.001 |
| MTMR2 | 1.661 | <0.01 | <0.001 |
| UBE2E2 | 1.682 | <0.01 | <0.001 |
| ART3 | 2.936 | <0.01 | <0.001 |
| SLC39A14 | 1.654 | <0.01 | <0.001 |
| LGALS7B | 0.664 | <0.01 | <0.001 |
| BOD1L1 | 1.388 | <0.01 | <0.001 |
| CDCA7L | 2.079 | <0.01 | <0.001 |
| MKI67 | 1.489 | <0.01 | <0.001 |
| NPTX2 | 0.780 | 0.011 | <0.001 |
| IFNAR2 | 1.525 | 0.013 | <0.001 |
| DCXR | 0.750 | 0.013 | <0.001 |
| PRC1 | 1.975 | 0.013 | <0.001 |
| GDI2 | 1.549 | 0.020 | <0.001 |
| WDR37 | 1.359 | 0.024 | <0.001 |
| TP63 | 0.594 | 0.028 | <0.001 |
| ENY2 | 1.812 | 0.028 | <0.001 |
| GGCT | 2.087 | 0.028 | <0.001 |
| MAP7D3 | 1.759 | 0.030 | <0.001 |
| TMA16 | 1.740 | 0.033 | <0.001 |
| TCF7L2 | 2.188 | 0.033 | <0.001 |
| SLC25A17 | 0.767 | 0.043 | <0.001 |
| BCAT2 | 0.666 | 0.048 | <0.001 |
| CDK19 | 1.548 | 0.048 | <0.001 |
| LZTFL1 | 1.517 | 0.049 | <0.001 |
| MYC | 1.311 | 0.304 | 0.008 |
